# Optimization of Patient Flow in Urgent Care Centers Using a Digital Tool for Recording Patient Symptoms and History: Simulation Study

**DOI:** 10.2196/26402

**Published:** 2021-05-21

**Authors:** Maryam Montazeri, Jan Multmeier, Claire Novorol, Shubhanan Upadhyay, Paul Wicks, Stephen Gilbert

**Affiliations:** 1 Ada Health GmbH Berlin Germany

**Keywords:** symptom assessment app, discrete event simulation, health care system, patient flow modeling, patient flow, simulation, urgent care, waiting times

## Abstract

**Background:**

Crowding can negatively affect patient and staff experience, and consequently the performance of health care facilities. Crowding can potentially be eased through streamlining and the reduction of duplication in patient history-taking through the use of a digital symptom-taking app.

**Objective:**

We simulated the introduction of a digital symptom-taking app on patient flow. We hypothesized that waiting times and crowding in an urgent care center (UCC) could be reduced, and that this would be more efficient than simply adding more staff.

**Methods:**

A discrete-event approach was used to simulate patient flow in a UCC during a 4-hour time frame. The baseline scenario was a small UCC with 2 triage nurses, 2 doctors, 1 treatment/examination nurse, and 1 discharge administrator in service. We simulated 33 scenarios with different staff numbers or different potential time savings through the app. We explored average queue length, waiting time, idle time, and staff utilization for each scenario.

**Results:**

Discrete-event simulation showed that even a few minutes saved through patient app-based self-history recording during triage could result in significantly increased efficiency. A modest estimated time saving per patient of 2.5 minutes decreased the average patient wait time for triage by 26.17%, whereas a time saving of 5 minutes led to a 54.88% reduction in patient wait times. Alternatively, adding an additional triage nurse was less efficient, as the additional staff were only required at the busiest times.

**Conclusions:**

Small time savings in the history-taking process have potential to result in substantial reductions in total patient waiting time for triage nurses, with likely effects of reduced patient anxiety, staff anxiety, and improved patient care. Patient self-history recording could be carried out at home or in the waiting room via a check-in kiosk or a portable tablet computer. This formative simulation study has potential to impact service provision and approaches to digitalization at scale.

## Introduction

### Background

Crowding in health care facilities occurs when the number of patients seeking care exceeds the care facility’s capacity in a given time period. Long queues of patients can lead to delayed care delivery, increased health risk for urgent cases, higher rates of hospital-borne infections, increased stress, and avoidable staff burden [1,2]. Crowding has also been associated with increased occurrence of preventable medical errors and with negative effects on clinical trial outcomes [3-5]. Many studies have shown that crowding in emergency departments (EDs) lowers satisfaction of patients [6], increases stress on staff, leads to less adherence of staff to guidelines, leads to less rapport between patients and health care professionals, and ultimately to a less “soft” interaction between patients and health care professionals [7]. Health care system performance can be measured in terms of patients’ waiting time and quality of the service, among other variables such as cost [8]. One method that can help analyze the performance of the whole system is patient flow modeling, which can aid decision-making in planning capacity, resources, and appointment scheduling [9].

Methods to improve the flow of health care delivery include eliminating unnecessary and duplicate activities, performing activities in parallel, and identifying alternative process flows [9]. History-taking and recording of patients’ symptoms by skilled health care professionals are often duplicated activities during triage and treatment in both urgent care centers (UCCs) and EDs [10].

Redundancy in data capture has been reported to reduce the quality of patient care [11], and a resultant practice recommendation was to take steps to resolve this issue. Similarly, a clinical study of randomly selected practices found that repetitive clinical notes can hinder coordination of patient care between health care professionals [12].

In the ED setting, a waiting room–based patient self-symptom and history-taking app (Ada Health, Germany) facilitated patient-to-health care professional communication, and triage nurses perceived this app as also having a workflow benefit by saving time [13]. The tool uses a probabilistic reasoning engine to collect demographic information, medical history, and symptoms. In a previous usability study, 97.8% (511/522) of patients found the symptom-taking system easy to use in the primary care waiting room [14]. A clinical vignette study showed that the system’s reasoning engine has similar levels of coverage, accuracy, and safety as human general practitioners [15], which is important for gathering comprehensive primary care histories. Symptom-taking and assessment tools from other providers have been judged by patients to provide useful diagnostic advice and to be easy to use [16,17]. However, the potential workflow benefits that might be experienced by using this tool in a more urgent setting remain unclear.

The term UCC can refer to several types of services, including walk-in centers, minor injury units, and urgent treatment centers, all with different levels of service [18-21]. As modeled in this study, a typical UCC is led by a physician (general practitioner), is open every day of the week, and is equipped to diagnose and treat common ailments. In the United Kingdom, this type of unit is known as an “urgent treatment center” [22]. Most prior research on triage, waiting, and consultation time distributions has been carried out in primary health clinics [23-25] or the ED [26-29]; thus, relatively little, with the exception of one study [30], has been reported for UCCs.

### System Simulation for Workflow Efficiency

We used a system simulation approach to understand the potential UCC flow and efficiency effects of a patient self-symptom and history-taking app. We specifically tested the hypothesis that waiting times and crowding in a UCC could be reduced through the introduction of a digital history-taking tool, and that system efficiency would be greater with use of the tool than through the addition of staff.

## Methods

### Simulation Development

We compared a scenario in which there was no patient self-system and history-taking tool to a scenario in which *every* patient entering the UCC waiting room had used the tool. Patient usage of the tool could be either: (i) at home (using a webpage or phone app); (ii) using check-in kiosks in a colocated ED waiting room, before fast-track redirection to the associated UCC; or (iii) using check-in kiosks at the UCC. In each case, it was modeled that the assessment report’s questions, answers, demographics, and symptoms would be transferred to the UCC’s electronic medical record system.

### Parameter Development: Clinical Setting

**Figure 1:**
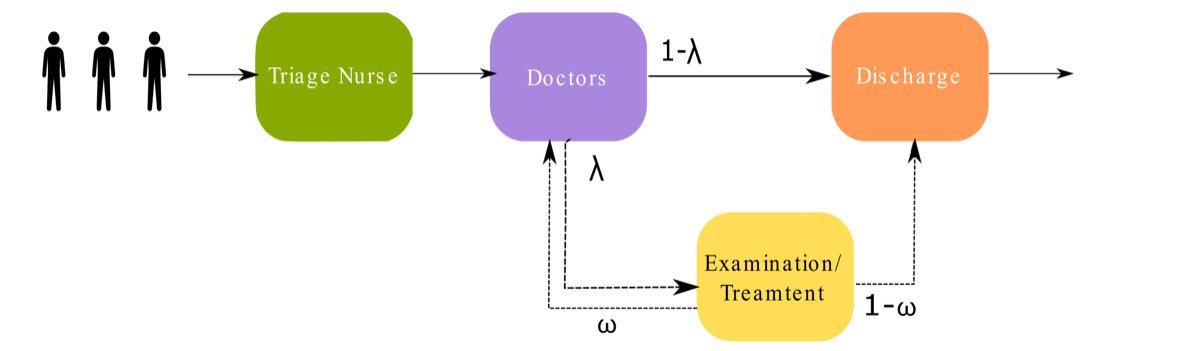
Illustration of the urgent care center, where patients arrive without any planned appointment. In the first step, a triage nurse runs a symptom assessment, and then patients are directed to the doctor. Depending on their situation, they may be examined/treated by another nurse and then discharged, sent back to a doctor, or discharged immediately by administrative staff.

We simulated a typical UCC in the first 4 hours of its opening. At the start of the patient journey (see Figure 1), a triage nurse assesses the symptoms of the patient. The patient then visits the doctor and then either visits the examination/treatment room (with probability λ) or is discharged (with probability 1 – λ). If a patient visits the examination/treatment room, they are either redirected to the doctor for further investigations (with probability ω) or discharged (with probability 1 – ω). Triage duration, consultation duration, number of staff in service, and arrival rate of the patients all affect the patient flow in the UCC. The baseline scenario of staffing of the UCC was taken from previous reports [18-21], and professional experience of one author (SU) and another colleague (Adel Baluch, Medical Director, Ada Health GmbH) who have each worked for over 5 years in National Health Service general practices, UCCs, and EDs. We assumed that there were two triage nurses, two doctors, one nurse for examination/treatment, and one administrator responsible for discharge (Table 1). We simulated the effects of the history-taking tool on queue size, waiting time for triage nurses, idle time, and utilization of triage nurses and doctors. Waiting times were modeled based on data collected from the ED setting [26,27].

**Table 1:** Baseline settings for simulation of the urgent care center.

Baseline setting	Triage nurse	Doctor	Examination/treatment nurse	Administration staff
Average duration of interaction with patient (minutes)	15	20	15	5
Number in service	2	2	1	1

### Parameter Development: Time Savings

Our model required a parameter for how much time could be saved through digital history-taking. A 2017 pilot implementation of a symptom app assessment in a busy (10,000 patients) UK primary care practice saved an estimated 1.9 minutes, as reported by doctors from over 300 primary care consultations (personal communication of unpublished pilot report, Dr. Vishaal Virani, Business Development & Client Success Director, Ada Health GmbH). A 2019 pilot involving structured interviews with 5 ED clinicians who viewed the handover report produced by the app estimated a time saving between 4 and 6 minutes (personal communication by email of unpublished product development data, Joseph Wolanski, Ada Health GmbH). Finally, in an observational study, time savings in the ED were estimated in the range of 2.5-5 minutes by triage nurses and physicians [13]. Based on these data, a range of potential time savings were modeled in this study.

### Setting Model Parameters: Crowding

First, we simulated the flow with different arrival rates to cause crowding, defined as more than 5 patients waiting for staff. We simulated the patient trajectory starting with an arrival rate of 0.1 patients per minute. Solutions were found to reach stability after 5000 simulations.

An arrival rate of 0.2 patients per minute (ie, one new patient every 5 minutes) was used, as described in further detail in Multimedia Appendix 1.

To explore our hypothesis that crowding can be reduced through the addition of a digital tool, we simulated *what-if* scenarios. Alongside this, we varied staffing from the baseline settings, as our hypothesis recognized that crowding can also likely be reduced by provision of more staff. We measured queue status, waiting time for the triage nurse, idle time, and utilization of triage nurses and doctors. Waiting time was defined as the interval between patient readiness for nurse triage and the end of the triage consultation, excluding the consultation duration. Idle time was defined as the period when one or more health care professionals is unoccupied. Utilization was defined as the ratio of the time the health care professionals are occupied to the total simulated time. Based on the previous study [13], we used a range of time savings by the app for triage (2.5, 3, 3.5, 4, 4.5, and 5 minutes) and for consultation (1.5, 2, 2.5, 3, and 3.5 minutes) to parameterize the model. We simulated 33 scenarios, including the baseline setting.

### Statistical Analysis

We used the package *Simmer* (version 4.4.0) [31], a process-oriented and trajectory-based discrete-event simulation (DES) package for R. Measures are reported after 5000 simulation runs as the overall mean and 95% CI, with the exception of utilization, which is reported as the median and IQR (as is standard in the DES package). The baseline case scenario was a UCC staffed with two triage nurses, two doctors, one treatment nurse, and one administrator responsible for discharge. We assumed that patient arrivals, triage, consultation, and discharge (all events in the patient flow through the UCC) follow a Poisson distribution and therefore the time interval distribution between all events follow exponential distributions. This approach stochastically models the variable duration of each event, including the variable patient-to-patient time-saving potential of the symptom and history report.

## Results

### Effect of Additional Staff

Table 2 shows how different staffing scenarios and use of the symptom and history-taking app could alter crowding.

**Table 2:** Effect of adding extra staff or using a digital symptom and history-taking app on queue sizes, idle time, and utilization of staff members, and patient waiting time for the triage nurse.

Scenario	Queue size for triage nurses (number of patients), mean (95% CI)	Queue size for doctors (number of patients), mean (95% CI)	Idle time of triage nurses (minutes), mean (95% CI)	Idle time of doctors (minutes), mean (95% CI)	Utilization of triage nurses (%), median (IQR)	Utilization of doctors (%), median (IQR)	Waiting time for triage nurses (minutes), mean (95% CI)
Baseline	8.47 (8.44-8.49)	5.44 (5.42-5.46)	13.99 (13.50-4.50)	24.10 (23.40-24.80)	96.9 (92.8-98.9)	93.3 (86.9-97.1)	34.05 (33.90- 34.21)
Baseline + triage nurse	3.4 (3.37-3.39)	9.53 (9.51-9.56)	61.04 (59.86-62.22)	13.43 (13.00-13.86)	40.5 (24.9-62.9)	96.1 (93.3-98.3)	13.2 (13.13- 13.28)
Baseline + triage nurse + doctor	3.47 (3.46-3.48)	5.57 (5.55-5.58)	59.78 (58.63-60.94)	40.34 (39.55-41.13)	47.3 (31.3-66.2)	90.2 (84.8-94.5)	13.54 (13.46- 13.62)
Baseline + digital tool (assuming minimum time saving)	6.29 (6.27-6.31)	6.82 (6.80-6.84)	22.74 (22.05-23.43)	19.32 (18.72-19.92)	94.4 (88.4-98.2)	94.4 (89.5-97.6)	25.44 (25.32- 25.56)
Baseline + digital tool (assuming maximum time saving)	3.84 (3.82-3.85)	8.2 (8.17-8.22)	41.73 (40.80-42.6)	17.04 (16.54-17.53)	88.0 (79.1-94.7)	95.1 (90.5-97.9)	15.5 (15.48- 15.64)

**Figure 2:**
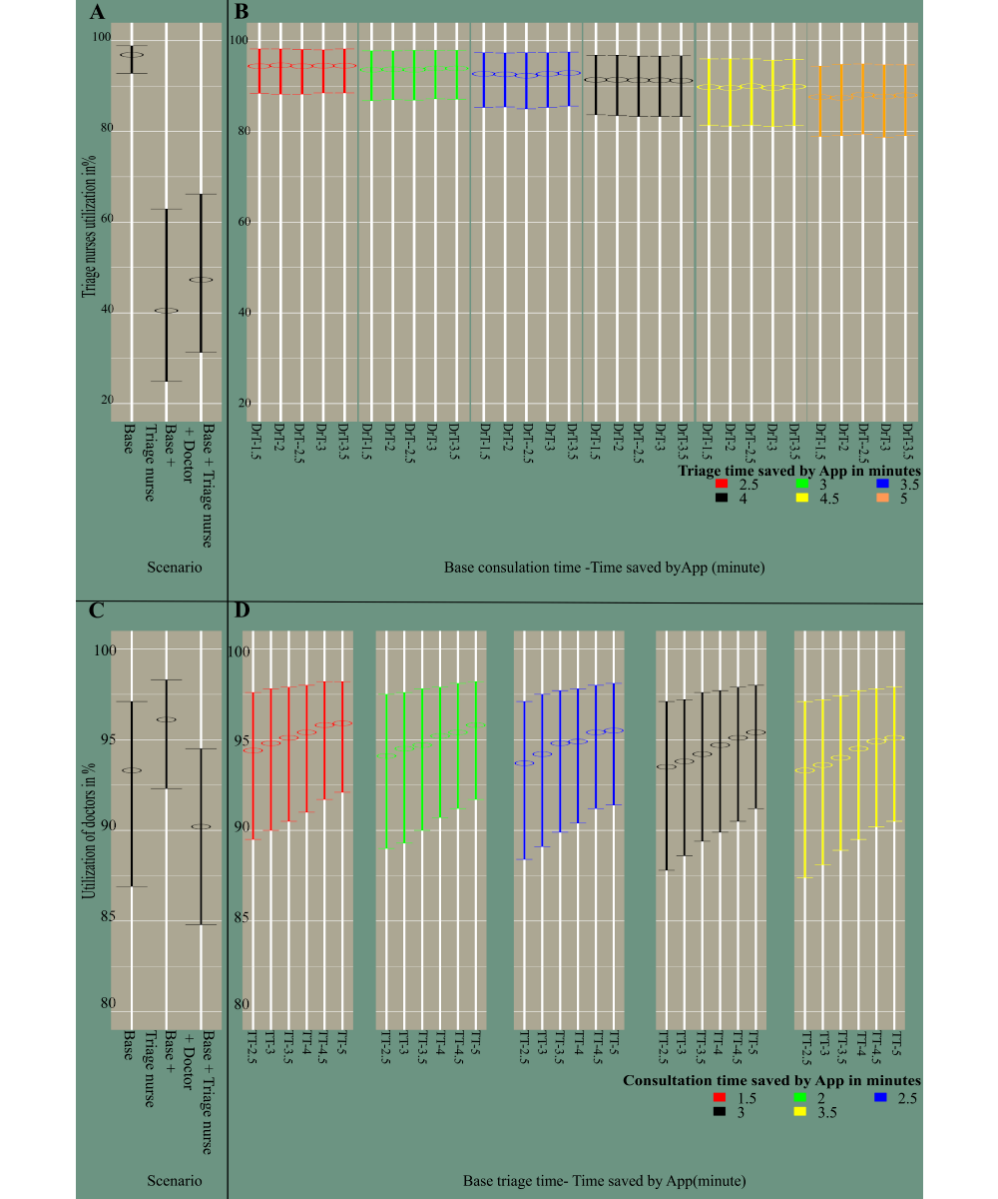
Utilization of triage nurses (A and B) and doctors (C and D) in percentage. A and C represent the idle time of the staff in scenarios where no app is used, which is the baseline case setting, and in scenarios with extra staff. B and D indicate the idle time of triage nurses and doctors in 30 different scenarios combining different time savings by the app in the triage and consultation processes. In B, the X-axis labels show triage time as the baseline triage time (TT) subtracted by time saved by the app (2.5, 3, 3.5, 4, 4.5, and 5 minutes). In D, the X-axis labels show the consultation time as the baseline doctor’s consultation time (DrT) subtracted by the time saved by the app (1.5, 2 ,2.5, 3, and 3.5 minutes).

Addition of an extra nurse reduced the queue length for triage nurses by around 60% but led to an approximately 75% increase in the queue length for the doctors. Providing one additional doctor could reduce the number of patients waiting for doctors to a similar situation as the baseline case (see Figure S1 in Multimedia Appendix 1). Adding one extra triage nurse resulted in a 336% increase of triage nurses’ idle time and a 44% decrease of the doctor’s idle time as more patients would be transferred to consultation in a shorter time (see Figure S2 in Multimedia Appendix 1). Adding one extra doctor led to a 67% increase of the mean idle time of doctors (Figure S2 in Multimedia Appendix 1). In the baseline case, the median triage nurses’ utilization was 96.9% and adding one extra triage nurse reduced this value to 40.5% (Figure 2). The median utilization of doctors was consistently maintained at a level of 90% or above. Adding a triage nurse led to a 61.23% reduction in the average waiting time for triage nurses (see Figure S3 in Multimedia Appendix 1).

### Effect of the Symptom and History-Taking App

Figure S1 in Multimedia Appendix 1 shows the impact of applying the symptom and history-taking app on the queue sizes of triage nurses and doctors in comparison to the baseline setting and the addition of extra staff. For the scenario where the time saved per patient by the app was modeled as 5 minutes, the time-saving impact of the app was equivalent to adding one triage nurse. Even when the app consultation time saving was modeled as 2.5 minutes per patient, this reduced patient queue length for triage by 25.73%. However, when nurse-led triage took less time per patient, the rate of flow to doctors increased, with a consequent increase in doctors’ queue size (see Table S1 in Multimedia Appendix 2).

### Idle and Utilization Time of Triage Nurses and Doctors

Longer triage times and shorter consultation times led to longer idle times for the doctors (Figure S2 in Multimedia Appendix 1). Assuming maximum app time saving, the triage nurses’ idle time almost doubled, whereas the doctors’ idle time was reduced by 30%. By contrast, assuming minimum app time saving, the average triage nurses’ idle time increased by about 62% and the average doctors’ idle time reduced by less than 20% (Table 2 and Table S2 in Multimedia Appendix 2). Median triage nurses’ utilization dropped only moderately, by about 9%, when modeling maximum app time savings. For minimum modeled app time saving, this drop in utilization was only 2.5%. Conversely, the median utilization of doctors was less dependent on the amount of time saved and was always above 93% (Figure 2, Table 2, Table S3 in Multimedia Appendix 2).

### Waiting Time for Triage Nurses

The more time saved by the app, the less time the patient needed to wait for a triage nurse (Figure S3 in Multimedia Appendix 1). When maximum app time saving was modeled, waiting time for a triage nurse dropped by 54.88%. (Table 2 and Table S4 in Multimedia Appendix 2). When minimal app time saving was modeled, the waiting time for triage dropped by 25.28%.

## Discussion

### Principal Findings

We simulated patient flow of a UCC in three conditions: (a) baseline, (b) with extra staff, and (c) with a digital symptom-taking tool. The shortest queue size and waiting time for triage nurses were achieved with the provision of one extra triage nurse (ie, a total of three triage nurses) and one additional doctor (ie, a total of two doctors). However, this approach may not be feasible due to limitations of available staff and high costs. Therefore, we hypothesized that use of a symptom and history-taking app before the triage process could be another possible solution. These apps have the potential to improve the patient flow in health care facilities such as hospitals, primary clinics, EDs, and UCCs [7], where a long queue of patients not only places substantial pressure on the health care workers but also on patients.

Our results suggest that for all measured variables, the amount of time saved by the app is an important determinant of the patient waiting time and system efficiency improvement. We found an amplification of time efficiency, through which relatively modest time savings per patient consultation accrued into substantial reduction in queuing time overall. The shortest modeled time saving from the app (2.5 minutes per patient) reduced the patient waiting time for triage by 25.28% and the longest time modeled from the app (5 minutes per patient) led to a 54.88% reduction in patient waiting time for a triage nurse.

Although crowding can also be resolved by additional staff, the simulation suggested that simply adding triage nurses may be inefficient as additional staff are only required at the busiest times. A digital symptom tool that could save 5 minutes per patient led to a reduction in waiting time equivalent to employing one extra triage nurse. Adding a triage nurse would have lowered staff utilization from 88% to 40%.

### Simulation for Improving Health System Efficiency

Simulation is an accepted and powerful method for hypothesis generation for the effects of new health care interventions on overall system efficiency. Simulation methods such as system dynamics, agent-based simulation, and discrete event simulation have gained substantial attention as helpful methods to tackle the complexities of analysis of patient flow in different areas. These applications include: (i) the detection of bottlenecks of the patient flow in health care facilities, (ii) optimizing flow management strategies such as scheduling and resource allocation rules, and (iii) estimating treatment cost in terms of the lengths of stay of patients [9,32,33]. Results of many simulation-based studies have already been implemented in real-world settings for better management of patient flow. One example evaluated scheduling, process flow, and resource levels in an oncology center [34], where the implementation of the changes proposed by the simulations resulted in improvement of the center’s system-wide performance. Another example applied the techniques explored here to a military outpatient primary care clinic. Simulation revealed a hybrid appointment/walk-in model for improving patient flow and optimized care provider utilization [35]. A final example applied a simulation model to identify factors contributing to flow blockage in an outpatient clinic, and its application led to significant improvements in real-life patient waiting time and physician utilization [36].

### Comparisons to the Wider Literature

One of the principal reasons that patients choose to go to a UCC is that they perceive waiting times to be lower than those experienced in general practitioner clinics or in the ED [37]. However, we were unable to identify any time-series studies that reported waiting times or other clinical processes in UCCs, and there has been little systematic data gathering on UCC clinical efficiency [18]. There is more substantial health service delivery and clinical efficiency research for the ED setting [38]; although time-series studies have been performed, the length of recording clinical history and symptoms, and how much time can be saved through digital history-taking tools have not been reported with certainty. We found no studies investigating the benefit or performance of self-assessment with a digital assessment tool in the UCC; however, some studies have reported the potential of self-triage for optimizing flow in subsections of EDs or in primary care units. Investigation of a bilingual self-triage kiosk in a pediatric ED showed that the system enabled parents to provide symptoms and history faster and more accurately than routine nurse-led triage [39].

### Unanswered Questions and Future Research

It is widely recognized that many promising digital innovations in health care are ultimately not adopted in practice, or are abandoned soon after limited local pilot utilization [40]. Often, it is not the limitations of the technology or difficulties in implementation that ultimately determine the success of the pilots and wider adoption, but rather the dynamic interactions between many of these factors [41]. This study explored the potential effects of a patient digital symptom and history-taking tool on patient flow and queuing, but did not explore the wider implications of the technology for the quality-of-care delivery, patient experience, patient safety, or the working experience of health care staff. These interlinked phenomena will be addressed in future studies.

ED crowding is mainly caused by patients who do not require urgent treatment [5] but whose medical history must be documented, accounting for approximately 41% of ED doctors’ time [10]. Crowding also leads to interruptions, which impair history-taking and documentation, particularly for inexperienced junior physicians who are overstretched [5].

Future research (including simulation studies, clinical investigations, and technology rollouts) should seek to understand the potential of such tools in reducing documentation burden, facilitating fast tracking, increasing patient safety, improving documentation accuracy, and ultimately reducing crowding.

### Strengths and Limitations

We used DES to simulate a queue of events. The choice of modeling technique, model structure, and parameter values limited the generalizability of the results as the nature of UCCs varies substantially [18]. In our model, we only considered a UCC without any planned appointments. We also assumed a first-in-first-out flow, irrespective of the urgency of treatment of individual patients. Patients and staff were all treated as passive, and we did not consider any ongoing learning that can influence patient and health worker interactions. We also assumed that there were enough digital devices available such that digital symptom assessment would not itself lead to another queue. We simulated 33 UCC setups, which were modified from a representative UCC baseline scenario (taken from experience and literature descriptions [18-21]) and cover a wide range of realistic UCC staffing scenarios. These 33 scenarios provide a balance between the range of real-world scenario coverage and study complexity, and also provide a reasonable basis of extrapolation of results. As UCCs vary substantially in terms of their staffing, resources, busyness, and layout, future studies should build on our results and simulate several real UCCs and include actual time measurements to substantiate the parameters.

We modeled under the assumption that the time spent taking history leads to a time saving for both the triage nurse and for the treating physician. One example from the literature highlights the level of duplication in a typical ED setting [10], where a history was taken by: (i) the triage nurse, (ii) clerking (student) physician, (iii) second clerking on transfer to the acute medical unit, (iv) at history review in the general ward round, and (v) at history retaking on admission to a specialty ward. In the ED setting, the retaking of clinical history provides no clinical benefit, with the history often recorded nearly verbatim to the previous history, as part of a recognized “futile clinical cycle” [10]. However, we acknowledge that in some cases, the histories taken by the triage nurse and the treating doctor have different purposes. We assumed that the information queried and the time spent in both cases overlapped to a large degree. Finally, we assumed that nurses and doctors could assess the recorded symptoms within their standard workflow.

### Conclusions

This simulation showed that even a small reduction in the time taken to assess symptoms can lead to a substantial reduction in the time patients wait for triage nurses, which could in turn lead to reduced patient anxiety, lower staff anxiety, and improved patient care. Compared to baseline, the use of a digital symptom-taking tool shortened the average patient waiting time to the same extent as adding an additional triage nurse to the UCC, with the additional advantages of higher staff efficiency. Such approaches have the potential to streamline service provision and accelerate approaches to digitalization in urgent care settings.

## Appendix

Multimedia Appendix 1 Supplementary methods and results.

Multimedia Appendix 2 Triage nurses' and doctors' queue sizes in 33 simulated scenarios (Table S1), triage nurses' and doctors' idle time in 33 simulated scenarios (Table S2), utilization of triage nurses and doctors in % (Table S3), and waiting time for the nurses in minutes (Table S4).

## Abbreviations

DES: discrete-event simulation
ED: emergency department
UCC: urgent care centre
