# Supplementary material for: Optimization of Patient Flow in Urgent Care Centers Using a Digital Tool for Recording Patient Symptoms and History: Simulation Study
Source: JMIR Form Res. 2021 May 21;5(5):e26402. doi: 10.2196/26402 (PMC8178735; doi:10.2196/26402)
Supplement: Multimedia Appendix 1 [file formative_v5i5e26402_app1.docx]

## Multimedia Appendix 1: Supplementary Methods and Results.

## Methods Supplement

### Setting Model Parameters – Arrival Rate and Crowding

For the arrival rate of 0.1 patients per minute, we observed an average queue size of 1.27 and 2.43 patients for triage nurse and doctors respectively. As we increased the patient arrival rate from 0.1 to 0.2 patients per minute, we observed an increase in nurse queue size from 1.27 to 8.46 patients and a corresponding increase in doctor queue size from 2.43 to 5.30. Therefore, for further analysis, we used the arrival rate of 0.2 patients per minute, or one new patient every 5 minutes.

## Results Supplement


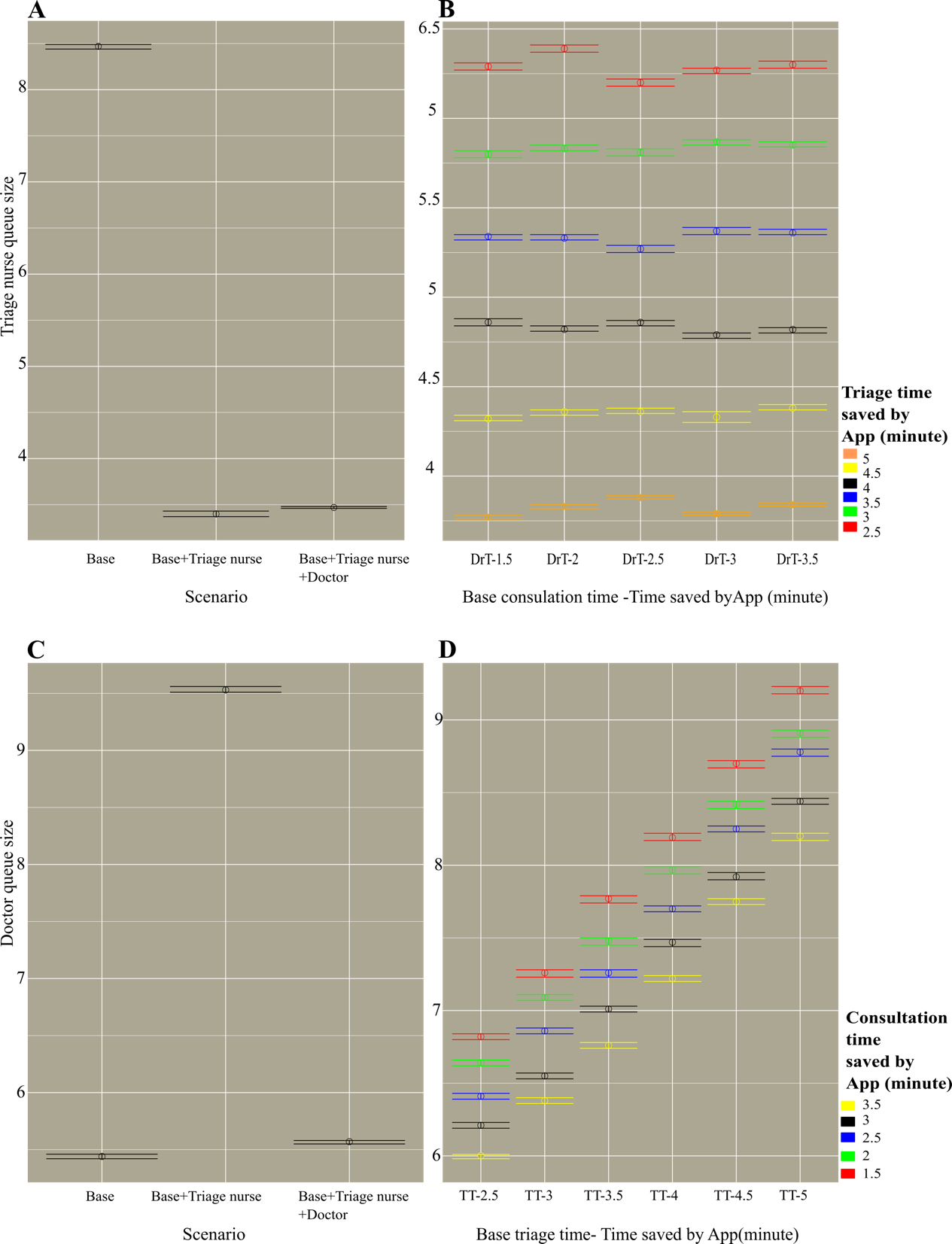


**Figure S1.** Queue size of triage nurses (A and B) and doctors (C and D). A and C represent the queues size of mentioned staff in scenarios where no App is used, which are base case setting and scenarios with extra staff. B and D on other hand, indicate queue sizes of triage nurses and doctors in 30 different scenarios of combining different time saving by app in triage and consultation process. In B, X axis labels show consultation time as a base doctor’s consultation time (DrT) subtracted by the time saved by App (1.5, 2 ,2.5 ,3 ,3.5 minutes). In D, X axis labels show triage time as a base triage time (TT) subtracted by time saved by App (2.5,3,3.5,4,4.5,5 minutes). Each circle represents the mean value of 5000 runs of simulation in each of 32 combinations. Horizontal lines represent 95%confidence intervals of corresponding average.


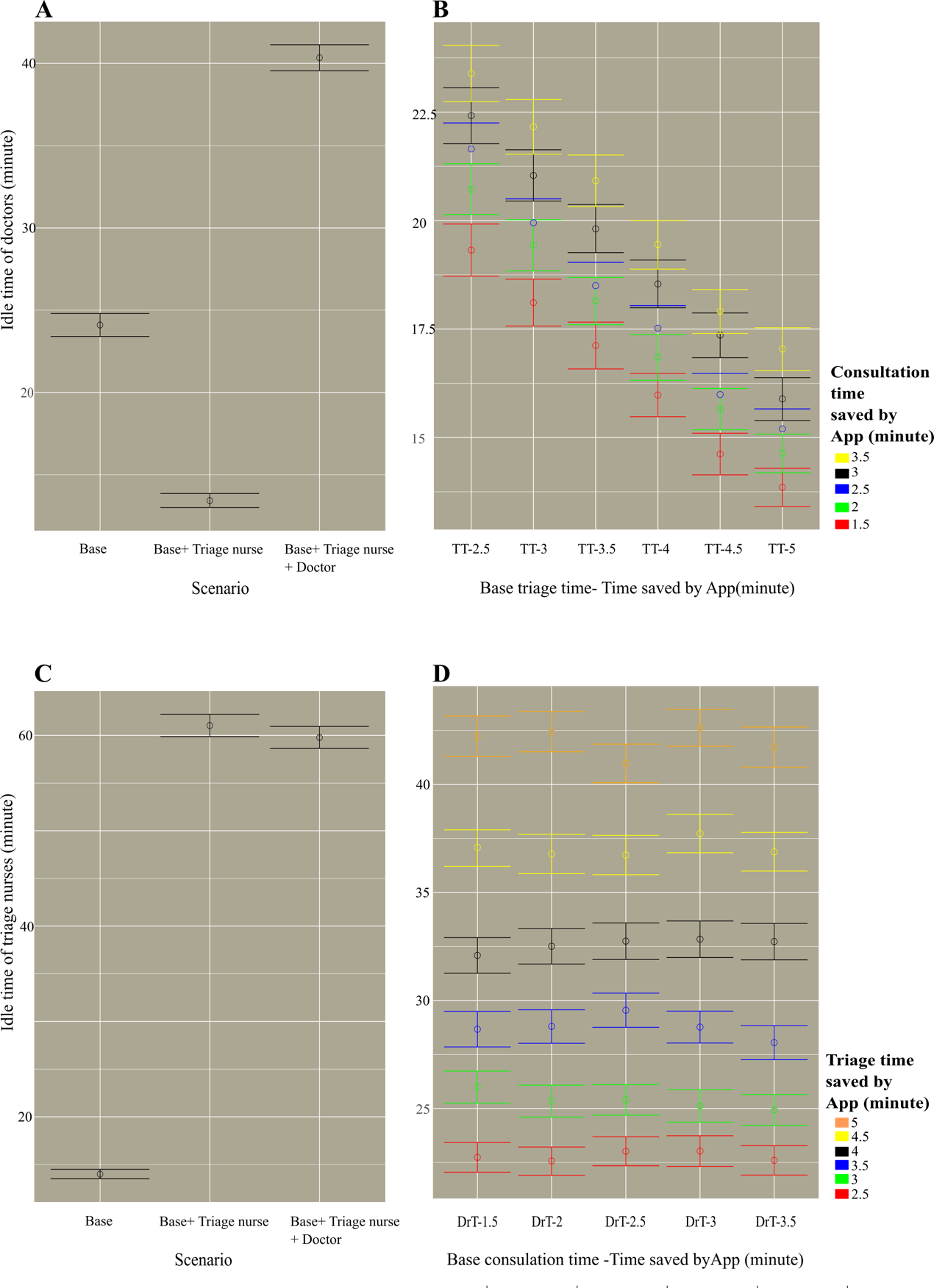


**Figure S2**. Idle time of doctors (A and B) and triage nurses (C and D). A and C represent the idle of mentioned staff in scenarios where no App is used, which are base case setting and scenarios with extra staff. B and D on other hand, indicate idle time of triage nurses and doctors in 30 different scenarios of combining different time saving by app in triage and consultation process. In B, X axis labels show triage time as a base triage time (TT) subtracted by time saved by App (2.5,3,3.5,4,4.5,5 minutes). In D, X axis labels show consultation time as a base doctor’s consultation time (DrT) subtracted by the time saved by App (1.5, 2 ,2.5 ,3 ,3.5 minutes). Each circle represents the mean value of 5000 runs of simulation in each of 32 combinations. Horizontal lines represent 95% confidence intervals of corresponding average.


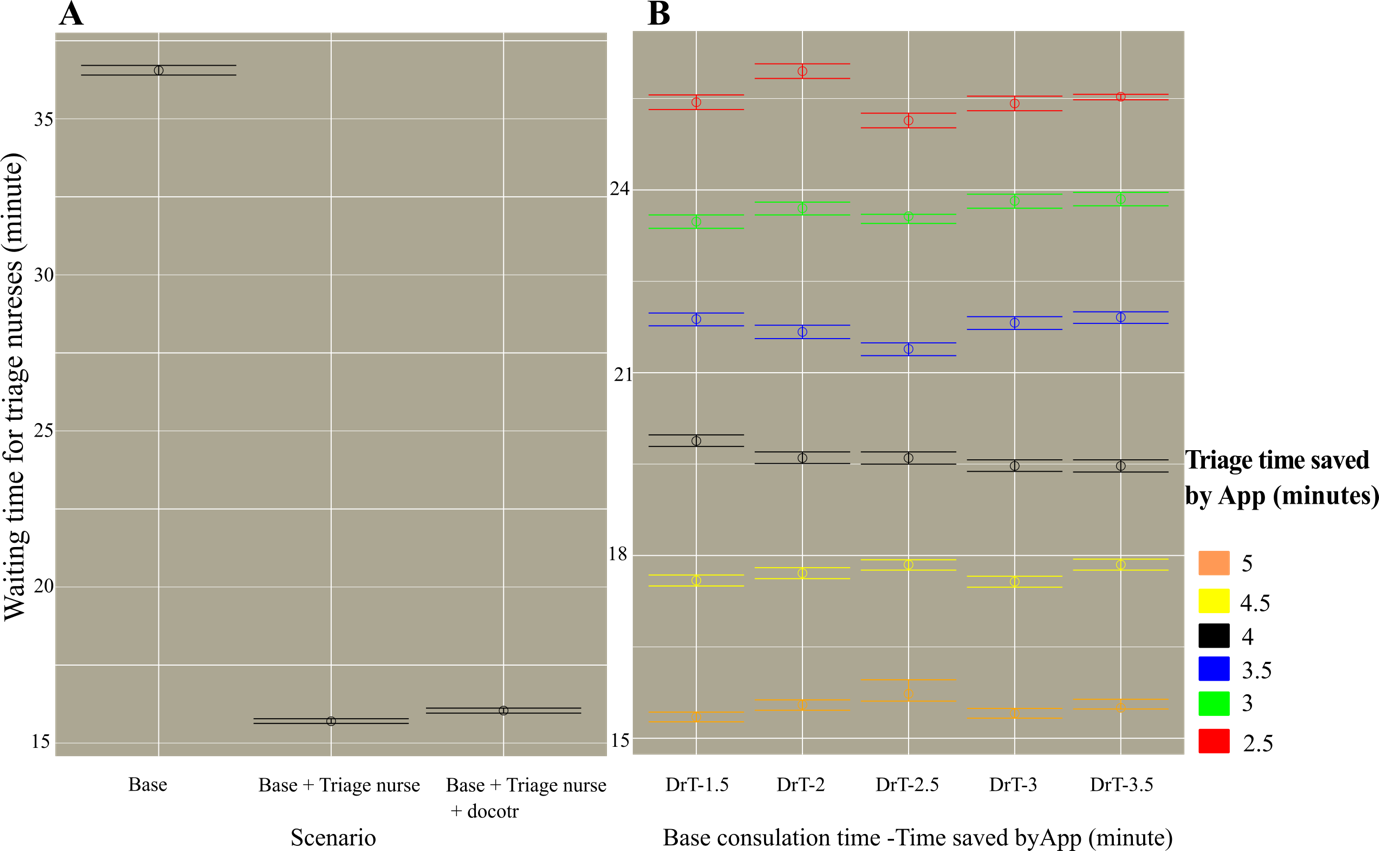


**Figure S3.** Waiting time for triage nurses in minutes. A represents the waiting time in scenarios where no App is used, which are base case setting and scenarios with extra staff. B on other hand, indicates waiting time in 30 different scenarios of combining different time saving by app in triage and consultation process. In B, X axis labels show consultation time as a base doctor’s consultation time (DrT) subtracted by the time saved by App (1.5, 2 ,2.5 ,3 ,3.5 minutes). Each circle represents the mean value of 5000 runs of simulation in each of 32 combinations. Horizontal lines represent 95% confidence intervals of corresponding average.
